# 

**Published:** 1908-12

**Authors:** 


**CONTENTS—DECEMBER, 1908.—VOL. XXIV, NO. 6.**

Criginal Articles—

Therapeutic Nihilism: Is It Ignorance or Egotism? By H. L. Henderson, M. D., Astoria, Oregon........................ 229

Some Practical Remarks Anent the Proposed Texas State Tuberculosis Sanitarium. By C. H. Wilkinson, M. D., Galveston, Texas .............................................. 240

Lymphatic Cyst of the Neck—Report of a Case. By Dr. C. S. Venable, San Antonio.................................... 245

Communications—

Proserpine Again .............................................. 247

Editorial Department—

Dr. Wainwright's Book........................................ 253

Dr. M. A. Taylor, Austin, Texas.............................. 254

Editorialets ................................................... 255

Abstracts and Selections...................................... 258

Publisher's Department ....................................... 272

